# Independent effects of the triglyceride-glucose index on all-cause mortality in critically ill patients with coronary heart disease: analysis of the MIMIC-III database

**DOI:** 10.1186/s12933-023-01737-3

**Published:** 2023-01-13

**Authors:** Rongting Zhang, Shanshan Shi, Weihua Chen, Yani Wang, Xueqin Lin, Yukun Zhao, Lihua Liao, Qian Guo, Xiaoying Zhang, Weiguo Li, Kaijun Zhang, Ying Liao, Yong Fang

**Affiliations:** 1 Department of Cardiology, Longyan First Affiliated Hospital of Fujian Medical University, Longyan, 364000 China; 2 The Graduate School of Clinical Medicine, Fujian Medical University, Fuzhou, 350000 China; 3 Department of Pulmonary and Critical Care Medicine, Longyan First Affiliated Hospital of Fujian Medical University, Longyan, 364000 China

**Keywords:** Triglyceride-glucose index, Insulin resistance, Coronary heart disease, All-cause mortality, MIMIC-III database

## Abstract

**Background:**

The triglyceride-glucose (TyG) index is a reliable alternative biomarker of insulin resistance (IR). However, whether the TyG index has prognostic value in critically ill patients with coronary heart disease (CHD) remains unclear.

**Methods:**

Participants from the Medical Information Mart for Intensive Care III (MIMIC-III) were grouped into quartiles according to the TyG index. The primary outcome was in-hospital all-cause mortality. Cox proportional hazards models were constructed to examine the association between TyG index and all-cause mortality in critically ill patients with CHD. A restricted cubic splines model was used to examine the associations between the TyG index and outcomes.

**Results:**

A total of 1,618 patients (65.14% men) were included. The hospital mortality and intensive care unit (ICU) mortality rate were 9.64% and 7.60%, respectively. Multivariable Cox proportional hazards analyses indicated that the TyG index was independently associated with an elevated risk of hospital mortality (HR, 1.71 [95% CI 1.25–2.33] P = 0.001) and ICU mortality (HR, 1.50 [95% CI 1.07–2.10] P = 0.019). The restricted cubic splines regression model revealed that the risk of hospital mortality and ICU mortality increased linearly with increasing TyG index (P for non-linearity = 0.467 and P for non-linearity = 0.764).

**Conclusions:**

The TyG index was a strong independent predictor of greater mortality in critically ill patients with CHD. Larger prospective studies are required to confirm these findings.

**Supplementary Information:**

The online version contains supplementary material available at 10.1186/s12933-023-01737-3.

## Introduction

Coronary heart disease (CHD) remains the leading cause of disease burden globally, and an increasing number of people die prematurely because of CHD [1]. According to the American Heart Association, one American experiences a heart attack every 40 s, and more than 350,000 die from CHD every year [2]. Critically ill patients admitted to the intensive care unit (ICU) have complex conditions and various etiologies. A study has shown that patients admitted to an ICU often have CHD (46.8%) [3]. However, few studies have assessed the prognosis of critically ill patients with CHD.

Insulin resistance (IR), defined as a decrease in the efficiency of insulin in promoting glucose uptake and utilization, is a prominent characteristic of metabolic syndrome [4]. The triglyceride-glucose (TyG) index has become a simple surrogate marker for IR, which is a fundamental clinical feature of severe metabolic syndrome and a marker for a group of pathological conditions associated with systemic inflammation, endothelial dysfunction, oxidative stress, and prothrombotic states [5, 6]. Metabolic syndrome is a critical CHD risk factor associated with a twofold-greater risk of CHD mortality, according to a 13-year follow-up study [7]. In addition, an elevated TyG index has been strongly associated with poor coronary collateralization in patients with chronic total occlusion [8]. However, well-developed collateral circulation of the coronary artery can improve the survival and prognosis of patients with coronary artery disease [9]. Moreover, multi-vessel coronary artery disease is a type of CHD with a high risk of adverse events [5]. An elevated TyG index is associated with a significantly higher risk of multi-vessel coronary artery disease [10]. However, whether this association exists in critically ill patients with CHD, who have more severe pathophysiological conditions, remains unclear. Assessing whether the TyG index is an effective prognostic method for ICU patients with CHD may aid in identifying patients at high risk of all-cause mortality for closer monitoring or potential early intervention.

Therefore, the aim of the present study was to evaluate potential relationships between the TyG index and all-cause mortality in critically ill patients with CHD.

## Methods

### Study population

The present study was a retrospective observational study. Data for our analysis came from a publicly available Medical Information Mart for Intensive Care III (MIMIC-III) database. MIMIC-III is a large freely available database comprising information for patients admitted to critical care units at a large tertiary care hospital in Boston between June 1, 2001 and October 10, 2012 [11]. One author (YL) completed the National Institutes of Health’s web-based course Protecting Human Research Participants and obtained permission to access the dataset. The database was approved for research use by the review committee of Massachusetts Institute of Technology and Beth Israel Deaconess Medical Center, and a waiver of informed consent was granted.

**Fig. 1:**
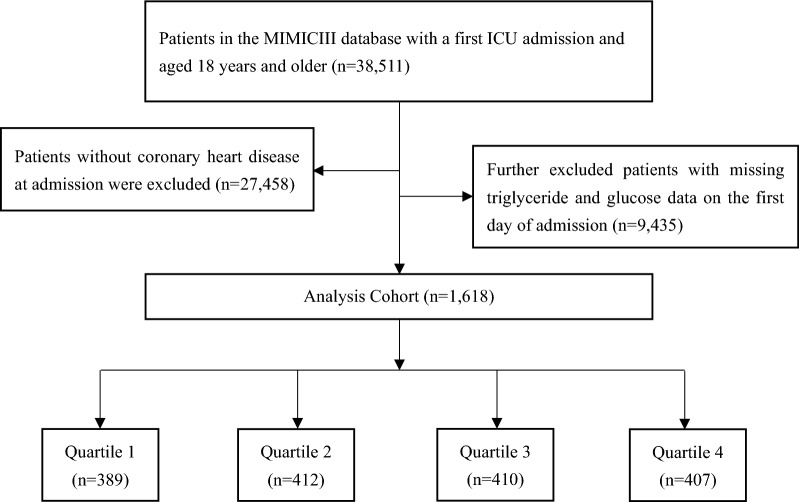
Flow of participants through the trial

We included 38,511 patients (≥ 18 years of age) admitted to the ICU in MIMIC-III, whereas patients without CHD at admission were excluded. Subsequently, we further excluded patients with missing triglyceride (TG) and glucose data on the first day of admission. A total of 1,618 patients were included in the final study cohort and divided into four groups according to the quartiles of the TyG index of the first day of the ICU stay (Fig. 1).

### Data collection

Structured Query Language (SQL) with PostgreSQL (version 9.6) was used to extract baseline characteristics, including sex, age, body mass index (BMI), and severity at admission (as measured by the Sequential Organ Failure Assessment (SOFA) score, Systemic inflammatory response syndrome (SIRS) score, Acute physiology score III (APSIII), Simplified acute physiological score II (SAPSII)), comorbidities, and laboratory variables within the first 24 h after ICU admission from the MIMIC-III database. The TyG index was calculated as ln [fasting TG (mg/dl) × fasting glucose (mg/dl)]/2 [12, 13]. CHD, heart failure, hypertension, atrial fibrillation, dyslipidemia, diabetes, respiratory failure, acute kidney injury (AKI), chronic kidney disease (CKD), and acute myocardial infarction (AMI) were defined with ICD-9 codes. The follow-up started from the date of admission and ended at death.

Variables with missing data are common in the MIMIC-III, single imputation was used to impute missing values. Variables with missing rate over 20% were converted to dummy variables in the models to avoid possible bias caused by direct filling missing values. All screening variables contained < 25% missing value (Additional file 1: Table S1).

### Primary outcome and clinical definition

The primary outcome of this study was in-hospital all-cause mortality, including hospital mortality and ICU mortality. CHD was defined as myocardial infarction (MI), acute coronary syndrome, ischemic heart disease, or percutaneous coronary intervention or coronary artery bypass grafting [14]. AKI was defined according to Kidney Disease: Improving Global Outcomes (KDIGO) guidelines as an increase in the serum creatinine (SCr) level by ≥ 0.3 mg/dL above baseline within 48 h [15].

### Statistical analysis

Continuous variables are presented as the mean ± standard deviation or median with interquartile range, and were compared with Student’s *t*-test. Categorical variables are presented as frequencies and percentages, and differences between groups were performed with a Pearson chi-square test or Fisher’s exact test. The associations between the TyG index and cardiovascular risk factors were assessed with Spearman’s rank correlation test or Pearson’s correlation test. To evaluate the incidence rate of primary outcome events among groups according to different levels of the TyG index, we used Kaplan–Meier survival analysis, and discrepancies among groups were evaluated with log-rank tests. We used Cox proportional hazards models to estimate the hazard ratio (HR) and 95% confidence interval (CI) between the TyG index and primary outcomes, and adjusted for multiple models. To avoid overfitting the model because of multicollinearity among variables, we also calculated the variance inflation factor. Variables with variance inflation factor ≥ 5 were excluded. Finally, clinically relevant and prognosis-associated variables were enrolled in the multivariate model: model 1: unadjusted; model 2: adjusted for age, sex, and BMI; model 3: adjusted for age, sex, BMI, dyslipidemia, hypertension, diabetes, CKD, respiratory failure, white blood cell (WBC), red blood cell (RBC), hemoglobin, SCr, and SIRS score. We used a restricted cubic splines model to examine the associations between the TyG index and outcomes. The TyG index was entered into the models as continuous variables and categorical variables (with the lowest TyG index value quartile as a reference group). The P values for trends were obtained through use of the quartile level as an ordinal variable. We further stratified analyses according to sex, age (≤ 65 and > 65 years), BMI (< 30 and ≥ 30 kg/m^2^), diabetes, hypertension, CKD, and AMI to identify the consistency of the prognostic value of the TyG index for primary outcomes. The interactions between TyG index and variables used for stratification were examined with likelihood ratio tests.

We used R 4.1.3 (R Foundation for Statistical Computing, Vienna, Austria) and SPSS 24.0 (IBM SPSS Statistics, Armonk, NY, USA) for data analysis. A two-sided *P-value* < 0.05 was considered statistically significant for all analyses.

## Results

A total of 1618 patients were finally enrolled in the present study. The mean age of the enrolled patients was 68.05 ± 14.05 years, and 1054 (65.14%) were men. The average TyG index value for all enrolled patients was 9.14 ± 0.71. The hospital mortality and ICU mortality rate were 9.64% and 7.60%, respectively (Table 1).

**Table 1:** Baseline characteristics of critical patients with CHD grouped according to TyG index quartiles^a^

Categories	Overall	Q1	Q2	Q3	Q4	*P-*value
	(N = 1618)	(N = 389)	(N = 412)	(N = 410)	(N = 407)	
Age, years	68.05 (14.05)	70.38 (14.50)	69.46 (14.08)	67.80 (13.72)	64.66 (13.28)	< 0.001
Male, n (%)	1054 (65.14)	262 (67.35)	264 (64.08)	266 (64.88)	262 (64.37)	0.762
BMI, kg/m^2^	29.60 (14.85)	29.03 (23.79)	28.99 (11.18)	28.86 (6.38)	31.40 (13.44)	0.180
SOFA score	3.00 (2.58)	2.62 (2.22)	2.71 (2.22)	3.21 (2.65)	3.43 (3.04)	< 0.001
SIRS score	2.43 (1.05)	2.27 (1.02)	2.35 (1.05)	2.53 (1.04)	2.55 (1.07)	< 0.001
APSIII	37.29 (17.14)	36.02 (15.26)	35.68 (15.60)	37.70 (17.51)	39.72 (19.55)	0.003
SAPSII	32.45 (12.62)	31.50 (11.55)	31.98 (11.50)	33.15 (13.03)	33.12 (14.15)	0.159
Comorbidities, n (%)						
Heart failure	651 (40.23)	143 (36.76)	158 (38.35)	165 (40.24)	185 (45.45)	0.067
Hypertension	866 (53.52)	195 (50.13)	228 (55.34)	220 (53.66)	223 (54.79)	0.455
Atrial fibrillation	401 (24.78)	114 (29.31)	100 (24.27)	101 (24.63)	86 (21.13)	0.064
Dyslipidemia	420 (25.96)	94 (24.16)	106 (25.73)	97 (23.66)	123 (30.22)	0.131
Diabetes	484 (29.91)	47 (12.08)	77 (18.69)	140 (34.15)	220 (54.05)	< 0.001
Respiratory failure	183 (11.31)	27 (6.94)	33 (8.01)	57 (13.90)	66 (16.22)	< 0.001
AKI^**b**^	830 (51.30)	181 (46.53)	182 (44.17)	228 (55.61)	239 (58.72)	< 0.001
CKD	179 (11.06)	43 (11.05)	38 (9.22)	45 (10.98)	53 (13.02)	0.390
AMI	1,126 (69.65)	264 (67.87)	289 (70.15)	282 (68.78)	291 (71.50)	0.698
PCI	1,127 (69.65)	261 (67.10)	286 (69.42)	292 (71.22)	288 (70.76)	0.588
CABG	186 (11.50)	41 (10.54)	35 (8.50)	64 (15.61)	46 (11.30)	0.013
Laboratory tests						
WBC, K/uL	11.76 (5.04)	10.91 (4.61)	11.33 (5.03)	11.88 (4.64)	12.87 (5.59)	< 0.001
Lymphocyte, %	15.24 (9.92)	15.69 (11.10)	15.37 (10.40)	14.72 (8.72)	15.25 (9.48)	0.699
Neutrophil, %	77.80 (12.16)	77.67 (13.16)	77.04 (12.71)	78.36 (10.68)	78.04 (12.16)	0.612
RBC, m/uL	4.17 (0.68)	4.10 (0.68)	4.18 (0.62)	4.18 (0.68)	4.23 (0.74)	0.040
Platelet, K/uL	250.27 (96.86)	245.36 (104.94)	249.52 (88.84)	252.96 (98.43)	253.00 (95.15)	0.645
Hemoglobin, g/dL	12.67 (2.07)	12.46 (2.01)	12.73 (1.95)	12.69 (2.13)	12.77 (2.15)	0.150
Serum potassium, mEq/L	4.22 (0.77)	4.15 (0.68)	4.22 (0.75)	4.18 (0.76)	4.34 (0.87)	0.004
Serum sodium, mEq/L	138.23 (4.00)	138.09 (4.52)	138.67 (3.53)	138.11 (3.81)	138.03 (4.07)	0.078
TC, mg/dL	161.20 (46.30)	148.67 (42.10)	161.24 (48.04)	165.06 (45.82)	169.44 (46.53)	< 0.001
TG, mg/dL	132.82 (89.64)	64.82 (22.25)	101.80 (28.19)	134.34 (45.68)	227.71 (119.13)	< 0.001
LDL, mg/dL	90.99 (39.95)	85.43 (35.95)	94.35 (42.71)	95.15 (39.97)	88.63 (40.04)	0.001
HDL, mg/dL	45.05 (14.21)	50.65 (15.15)	46.46 (13.66)	43.60 (13.38)	39.54 (12.27)	< 0.001
HbA1c, %	6.45 (1.42)	5.95 (0.98)	6.04 (0.79)	6.25 (1.19)	7.47 (1.85)	< 0.001
Glucose, mg/dL	165.39 (86.07)	134.58 (51.49)	146.48 (52.93)	166.13 (83.95)	213.46 (116.12)	< 0.001
Albumin, g/dL	3.49 (0.58)	3.48 (0.54)	3.48 (0.60)	3.50 (0.54)	3.49 (0.63)	0.967
Ucr, mg/dL	95.46 (65.61)	98.04 (66.63)	99.33 (60.41)	89.65 (62.52)	95.71 (72.06)	0.785
Scr, mg/dL	1.27 (1.11)	1.22 (1.21)	1.24 (1.02)	1.25 (1.00)	1.36 (1.19)	0.270
BUN, mg/dL	24.30 (15.87)	22.54 (12.96)	23.46 (14.17)	24.67 (16.45)	26.44 (18.92)	0.004
TyG index	9.14 (0.71)	8.27 (0.37)	8.88 (0.13)	9.33 (0.14)	10.06 (0.43)	< 0.001
Events						
LOS ICU, days	3.89 (5.80)	3.40 (6.03)	3.56 (5.28)	3.92 (4.62)	4.65 (6.96)	0.011
LOS Hospital, days	7.11 (7.70)	6.42 (7.10)	6.63 (7.08)	7.11 (6.22)	8.27 (9.81)	0.003
ICU mortality, n (%)	123 (7.60)	16 (4.11)	28 (6.80)	40 (9.76)	39 (9.58)	0.007
Hospital mortality, n (%)	156 (9.64)	20 (5.14)	35 (8.50)	50 (12.20)	51 (12.53)	0.001

*CHD* coronary heart disease, *TyG* index triglyceride glucose index, *BMI* body mass index, *SOFA* sequential organ failure assessment, *SIRS* systemic inflammatory response syndrome, *APSIII* acute physiology score III, *SAPSII* simplifed acute physiological score II, *AKI* acute kidney injury, *CKD* chronic kidney disease, *AMI* acute myocardial infarction, *PCI* percutaneous coronary intervention *CABG* coronary artery bypass grafting, *WBC* white blood cell, *RBC* red blood cell, *TC* total cholesterol, *TG* triglyceride, *LDL* low-density lipoprotein, *HDL* high-density lipoprotein, *HbA1c* hemoglobin A1c, *Ucr* urine creatinine, *Scr* serum creatinine, *BUN* blood urea nitrogen, *LOS* length of stay, *ICU* intensive care unit

^**a**^TyG index: Q1 (6.23–8.65), Q2 (8.65–9.10), Q3 (9.10–9.58), Q4 (9.58–11.78)

^**b**^AKI was defined according to KDIGO guidelines as an increase in serum creatinine (Scr) by ≥ 0.3 mg/dl (≥ 26.5 μmol/l) from baseline within 48 h

### Baseline characteristics

Baseline characteristics of the study patients according to the TyG index quartiles are presented in Table 1. Patients were divided into quartiles according to the admission TyG index levels (quartile [Q] 1: 6.23–8.65; Q2: 8.65–9.10; Q3: 9.10–9.58; Q4: 9.58–11.78). The mean levels of TyG index of the four groups were 8.27 ± 0.37, 8.88 ± 0.13, 9.33 ± 0.14 and 10.06 ± 0.43, respectively. Patients with higher TyG index were generally younger, higher severity of illness scores on admission, higher prevalence of diabetes, respiratory failure, AKI, higher levels of WBC, RBC, serum potassium, total cholesterol, LDL, HbA1c and blood urea nitrogen, and lower levels of HDL compared to the lower group. With increasing TyG index, ICU length of stay (3.40 days vs. 3.56 days vs. 3.92 days vs. 4.65 days, *P* = 0.011), hospital length of stay (6.42 days vs. 6.63 days vs. 7.11 days vs. 8.27 days, *P* = 0.003), ICU mortality (4.11% vs. 6.80% vs. 9.76% vs. 9.58%, *P* = 0.007), and hospital mortality (5.14% vs. 8.50% vs. 12.20% vs. 12.53%, *P* = 0.001) increased gradually.

Baseline characteristics between survivors and non-survivors are presented in Table 2. Patients in the non-survivor group showed higher age, and higher prevalence of heart failure, atrial fibrillation, respiratory failure, AKI, and CKD (*P* < 0.05). In terms of laboratory indicators, participants with an endpoint event had higher levels of WBC, neutrophils, serum potassium, fasting blood glucose (FBG), SCr, and blood urea nitrogen, but lower levels of lymphocytes, RBC, hemoglobin, total cholesterol, low-density lipoprotein (LDL), and albumin (*P* < 0.05). No significant difference was observed in sex, BMI, hypertension, dyslipidemia, diabetes, AMI, TG, high-density lipoprotein (HDL), and hemoglobin A1c (HbA1c) (*P* > 0.05). Patients in the non-survivor group had higher SOFA scores, SIRS scores, APSIII, and SAPSII than those in the survivor group. The TyG index levels in the non-survivor group were significantly higher than those in the survivor group (9.35 ± 0.73 vs. 9.12 ± 0.71, *P* < 0.001).

**Table 2:** Baseline characteristics of the Survivors and Non-survivors groups

Characteristic	Overall	Survivors	Non-survivors	*P-value*
	(N = 1618)	(N = 1462)	(N = 156)	
Age, years	68.05 (14.05)	67.31 (14.08)	74.97 (11.74)	< 0.001
Male, n (%)	1054 (65.14)	963 (65.87)	91 (58.33)	0.060
BMI, kg/m^2^	29.60 (14.85)	29.79 (15.50)	27.93 (6.37)	0.256
SOFA score	3.00 (2.58)	2.70 (2.26)	5.80 (3.50)	< 0.001
SIRS score	2.43 (1.05)	2.35 (1.04)	3.11 (0.92)	< 0.001
APSIII	37.29 (17.14)	35.03 (14.58)	58.44 (23.72)	< 0.001
SAPSII	32.45 (12.62)	30.72 (11.04)	48.68 (14.81)	< 0.001
Comorbidities, n (%)				
Heart failure	651 (40.23)	574 (39.26)	77 (49.36)	0.014
Hypertension	866 (53.52)	793 (54.24)	73 (46.79)	0.076
Atrial fibrillation	401 (24.78)	330 (22.57)	71 (45.51)	< 0.001
Dyslipidemia	420 (25.96)	386 (26.40)	34 (21.79)	0.212
Diabetes	484 (29.91)	436 (29.82)	48 (30.77)	0.806
Respiratory failure	183 (11.31)	119 (8.14)	64 (41.03)	< 0.001
AKI^a^	830 (51.30)	707 (48.36)	123 (78.85)	< 0.001
CKD	179 (11.06)	152 (10.40)	27 (17.31)	0.009
AMI	1126 (69.59)	1022 (69.90)	104 (66.67)	0.403
PCI	1127 (69.65)	1058 (72.37)	69 (44.23)	< 0.001
CABG	186 (11.50)	177 (12.11)	9 (5.77)	0.018
Laboratory tests				
WBC, K/uL	11.76 (5.04)	11.57 (4.91)	13.52 (5.80)	< 0.001
Lymphocyte, %	15.24 (9.92)	15.73 (10.09)	11.62 (7.73)	< 0.001
Neutrophil, %	77.80 (12.16)	77.43 (12.05)	80.48 (12.74)	0.006
RBC, m/uL	4.17 (0.68)	4.19 (0.68)	4.04 (0.66)	0.009
Platelet, K/uL	250.27 (96.86)	250.25 (95.37)	250.45 (110.24)	0.981
Hemoglobin, g/dL	12.67 (2.07)	12.73 (2.07)	12.08 (1.97)	< 0.001
Serum potassium, mEq/L	4.22 (0.77)	4.19 (0.75)	4.51 (0.88)	< 0.001
Serum sodium, mEq/L	138.23 (4.00)	138.26 (3.86)	137.94 (5.16)	0.346
TC, mg/dL	161.20 (46.30)	162.37 (45.83)	149.09 (49.49)	0.001
TG, mg/dL	132.82 (89.64)	133.13 (88.04)	129.92 (103.74)	0.670
LDL, mg/dL	90.99 (39.95)	91.97 (39.50)	80.79 (43.19)	0.002
HDL, mg/dL	45.05 (14.21)	45.08 (13.90)	44.74 (17.17)	0.790
HbA1c, %	6.45 (1.42)	6.46 (1.44)	6.31 (1.15)	0.413
Glucose, mg/dL	165.39 (86.07)	162.23 (84.14)	195.18 (97.85)	< 0.001
Albumin, g/dL	3.49 (0.58)	3.54 (0.54)	3.16 (0.70)	< 0.001
UCr, mg/dL	95.46 (65.61)	97.70 (66.32)	86.57 (62.36)	0.215
SCr, mg/dL	1.27 (1.11)	1.22 (1.08)	1.67 (1.31)	< 0.001
BUN, mg/dL	24.30 (15.87)	23.26 (15.17)	34.04 (18.78)	< 0.001
TyG index	9.14 (0.71)	9.12 (0.71)	9.35 (0.73)	< 0.001

*BMI* body mass index, *SOFA* sequential organ failure assessment, *SIRS* systemic inflammatory response syndrome, *APSIII* acute physiology score III, *SAPSII* simplifed acute physiological score II, *AKI* acute renal injury, *CKD* chronic kidney disease, *AMI* acute myocardial infarction, *PCI*, percutaneous coronary intervention, *CABG*, coronary artery bypass grafting, *WBC* white blood cell, *RBC* red blood cell, *TC* total cholesterol, *TG* triglyceride, *LDL* low-density lipoprotein, *HDL* high-density lipoprotein, *HbA1c* hemoglobin A1c, *UCr* urine creatinine, *SCr* serum creatinine, *BUN* blood urea nitrogen, *TyG* index triglyceride glucose index

^a^AKI was defined according to KDIGO guidelines as an increase in serum creatinine (Scr) by ≥ 0.3 mg/dl (≥ 26.5 μmol/l) from baseline within 48 h

### Correlation between the TyG index and cardiovascular risk factors

As shown in Table 3, the TyG index was significantly associated with traditional or commonly used risk factors for cardiovascular disease (CVD). A positive correlation was found between the TyG index and BMI, FBG, HbA1c, TG, total cholesterol, and LDL, whereas a negative correlation was observed with age and HDL.

**Table 3:** Correlations between the TyG index and traditional cardiovascular risk factors

Variable	Correlation coefficient	*P* value
Age	− 0.149	< 0.001
Sex, male	− 0.025	0.310
BMI	0.074	0.025
FBG	0.363	< 0.001
HbA1c	0.437	< 0.001
TG	0.760	< 0.001
TC	0.194	< 0.001
LDL	0.051	0.046
HDL	− 0.281	< 0.001
Uric acid	− 0.062	0.634
Serum creatinine	0.044	0.077

*BMI*, body mass index, *FBG* fasting blood glucose, *HbA1c* glycosylated hemoglobin A1c, *TG* triglyceride, *TC* total cholesterol, *LDL* low-density lipoprotein, *HDL* high-density lipoprotein

### Primary outcomes

**Fig. 2:**
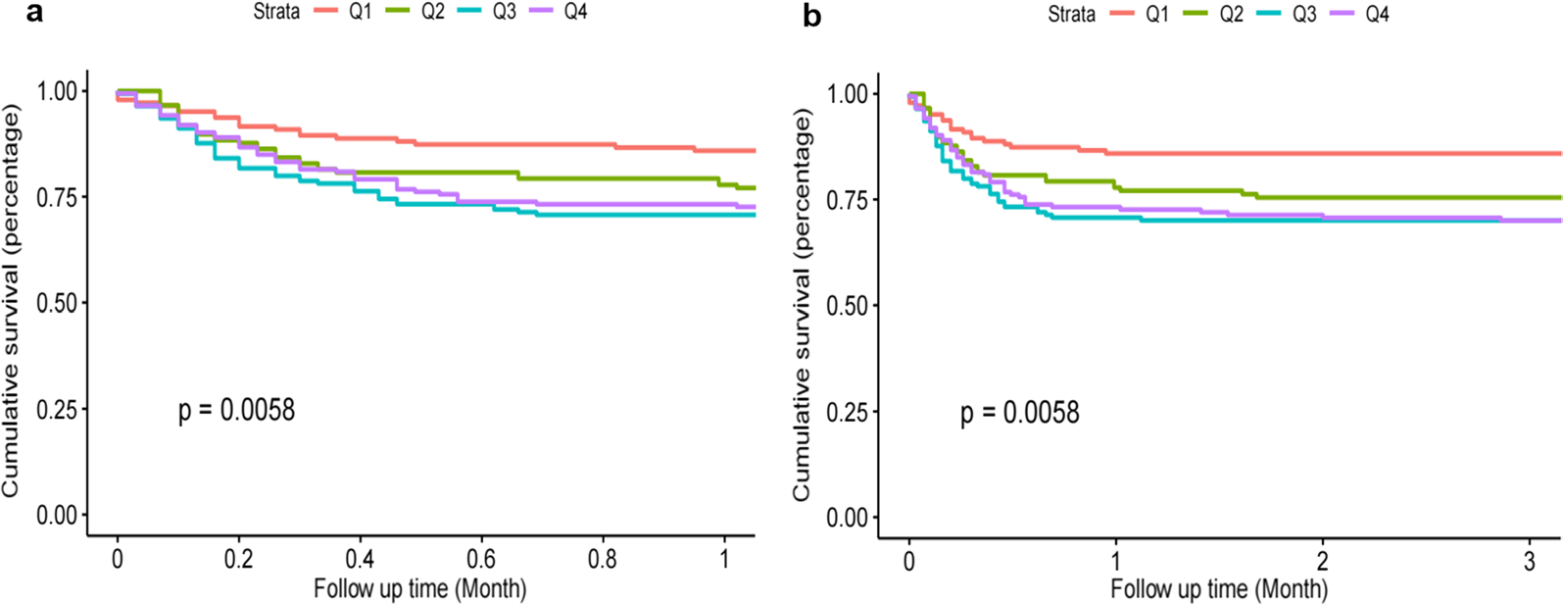
Kaplan–Meier survival analysis curves for all-cause mortality. Footnote TyG index quartiles: Q1 (6.23–8.65), Q2 (8.65–9.10), Q3 (9.10–9.58), Q4 (9.58–11.78). Kaplan–Meier curves showing cumulative probability of all-cause mortality according to groups at 1 month (**a**), and 3 months (**b**)

The Kaplan–Meier survival analysis curves for incidence of primary outcomes among groups, according to the TyG index quartiles are shown in Fig. 2. A statistically significant difference in mortality rate among groups was observed during the short-term follow-up of 1 month (log-rank *P* = 0.0058, Fig. 2a). A significant result was also observed during the 3 months of follow-up (log-rank *P* = 0.0058, Fig. 2b).

**Fig. 3:**
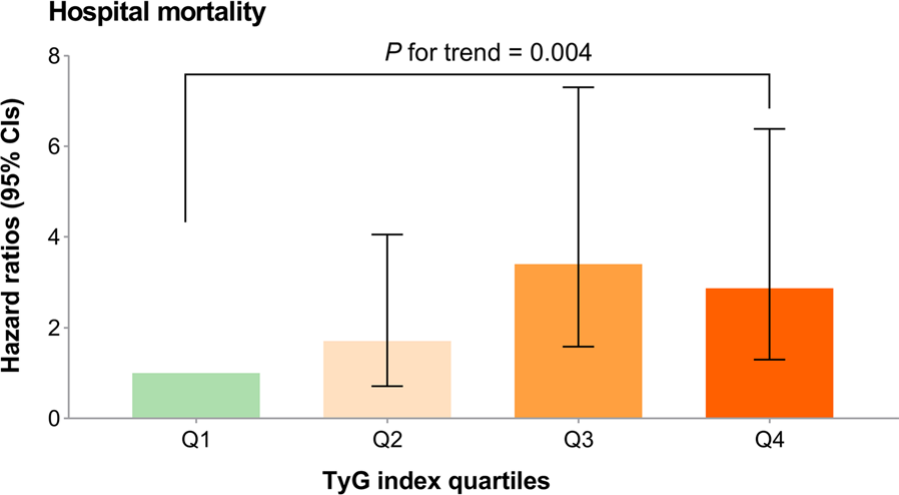
Hazard ratios (95% CIs) for hospital mortality according to TyG index quartiles after adjusting for age, sex, BMI, dyslipidemia, hypertension, diabetes, chronic kidney disease, respiratory failure, white blood cell, red blood cell, hemoglobin, serum creatinine, SIRS score. Error bars indicate 95% CIs. The first quartile is the reference. CIs, confidence intervals; TyG, triglyceride-glucose

**Fig. 4:**
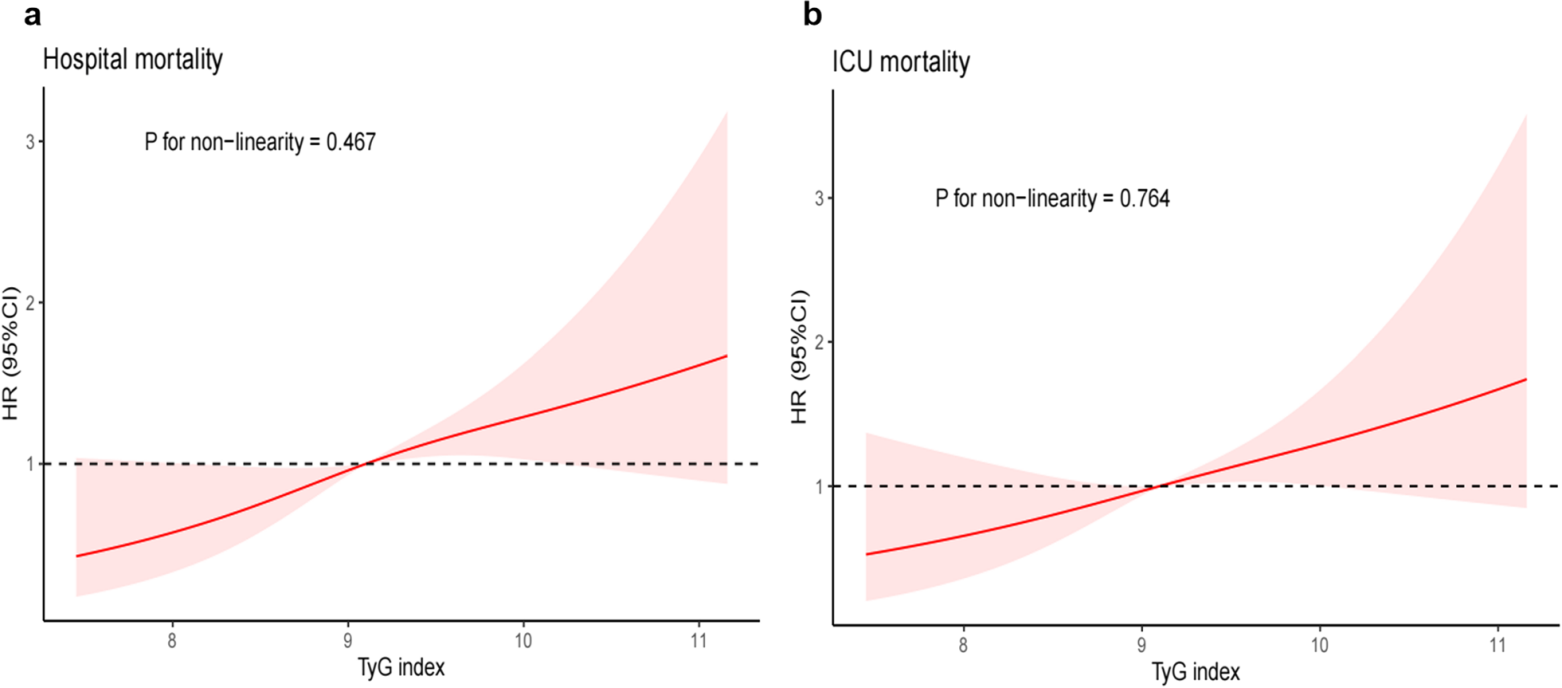
Restricted cubic spline curve for the TyG index hazard ratio. **a** Restricted cubic spline for hospital mortality. **b** Restricted cubic spline for ICU mortality. HR, hazard ratio; CI, confidence interval; ICU, intensive care unit; TyG, triglyceride-glucose

Cox proportional risk analysis indicated a significant association between TyG index and hospital mortality, in both the unadjusted model (HR, 1.39 [95%CI 1.13–1.71] *P* = 0.002) and the fully adjusted model (HR, 1.71 [95%CI 1.25–2.33] *P* = 0.001) when the TyG index was a continuous variable. Furthermore, when the TyG index was a nominal variable, it was also associated with hospital mortality in both an unadjusted model (Q1 vs. Q2: HR, 1.80 [95% CI 1.04–3.12] *P* = 0.036; Q3: HR, 2.33 [95% CI 1.39–3.92] *P* = 0.001; Q4: HR, 2.25 [95% CI 1.34–3.77] *P* = 0.002; *P* for trend = 0.003) and a fully adjusted model (Q1 vs. Q2: HR, 1.71 [95% CI 0.72–4.06] *P* = 0.223; Q3: HR, 3.41 [95% CI 1.59–7.31] *P* = 0.002; Q4: HR, 2.88 [95% CI 1.30–6.39] P = 0.009; *P* for trend = 0.004), and showed a tendency to increase with the TyG index (Table 4; Fig. 3). Similar results were obtained in multivariate Cox proportional risk analysis of the TyG index and ICU mortality (Table 4; Additional file 2: Figure S1). The restricted cubic splines regression model revealed that the risk of hospital mortality and ICU mortality increased linearly with increasing TyG index (*P* for non-linearity = 0.467 and *P* for non-linearity = 0.764, respectively) (Fig. 4).

**Table 4:** Cox proportional hazard ratios (HR) for all-cause mortality

Categories	Model 1			Model 2			Model 3		
	HR (95% CI)	*P-*value	*P *for trend	HR (95% CI)	*P-*value	*P *for trend	HR (95% CI)	*P-*value	*P *for trend
Hospital mortality									
Continuous variable per 1 unit	1.39 (1.13–1.71)	0.002		1.79 (1.35–2.38)	< 0.001		1.71 (1.25–2.33)	0.001	
Quartile ^**a**^			0.003			< 0.001			0.004
Q1 (N = 389)	Ref.			Ref.			Ref.		
Q2 (N = 412)	1.80 (1.04–3.12)	0.036		1.76 (0.75–4.15)	0.194		1.71 (0.72–4.06)	0.223	
Q3 (N = 410)	2.33 (1.39–3.92)	0.001		3.65 (1.73–7.71)	0.001		3.41 (1.59–7.31)	0.002	
Q4 (N = 407)	2.25 (1.34–3.77)	0.002		3.50 (1.63–7.53)	0.001		2.88 (1.30–6.39)	0.009	
ICU mortality									
Continuous variable per 1 unit	1.35 (1.07–1.71)	0.011		1.71 (1.26–2.32)	0.001		1.50 (1.07–2.10)	0.019	
Quartile			0.010			0.001			0.031
Q1 (N = 389)	Ref.			Ref.			Ref.		
Q2 (N = 412)	1.79 (0.97–3.31)	0.063		1.87 (0.72–4.86)	0.198		1.77 (0.68–4.62)	0.246	
Q3 (N = 410)	2.31 (1.29–4.13)	0.005		4.01 (1.74–9.28)	0.001		3.47 (1.48–8.16)	0.004	
Q4 (N = 407)	2.14 (1.19–3.83)	0.011		3.46 (1.46–8.17)	0.005		2.48 (1.01–6.07)	0.047	

Model 1: unadjusted

Model 2: adjusted for age, sex, BMI

Model 3: adjusted for age, sex, BMI, dyslipidemia, hypertension, diabetes, chronic kidney disease, respiratory failure, white blood cell, red blood cell, hemoglobin, serum creatinine, SIRS score

^**a**^TyG index: Q1 (6.23–8.65), Q2 (8.65–9.10), Q3 (9.10–9.58), Q4 (9.58–11.78)

**Fig. 5:**
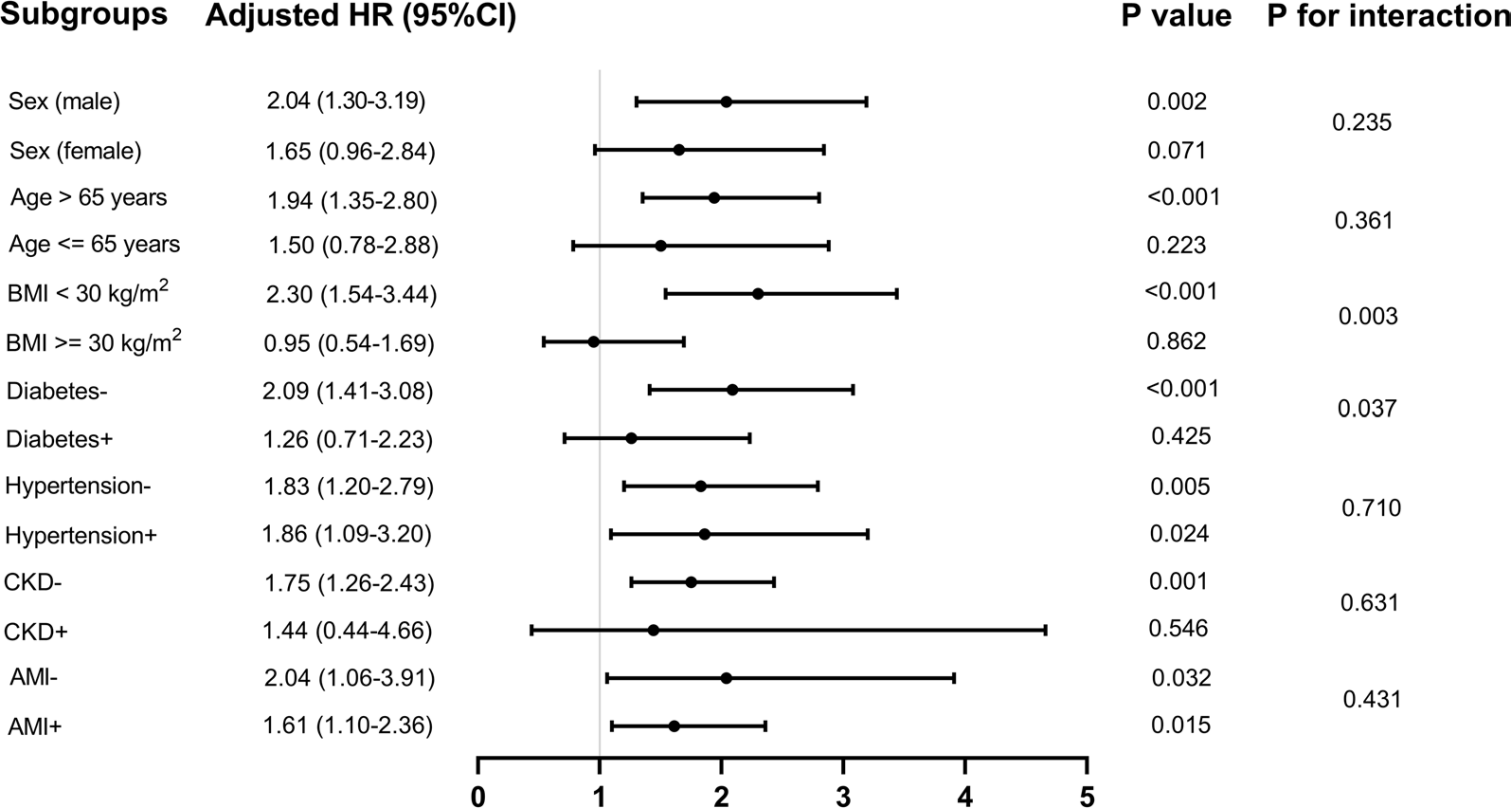
Forest plots of hazard ratios for the primary endpoint in different subgroups. HR, hazard ratio; CI, confidence interval; BMI, body mass index; CKD, chronic kidney disease; AMI, acute myocardial infarction

Further evaluation of the risk stratification value of the TyG index for primary outcomes was conducted in various subgroups of the study population, including sex, age, BMI, diabetes, hypertension, CKD, and AMI (Fig. 5). The TyG index was significantly associated with higher risk of hospital mortality in subgroups of male [HR (95% CI) 2.04 (1.30–3.19)], those aged > 65 years [HR (95% CI) 1.94 (1.35–2.80)], those with BMI < 30 kg/m^2^ [HR (95% CI) 2.30 (1.54–3.44)], those without diabetes [HR (95% CI) 2.09 (1.41–3.08)], those with hypertension [HR (95% CI) 1.86 (1.09–3.20)], those without hypertension [HR (95% CI) 1.83 (1.20–2.79)], those without CKD [HR (95% CI) 1.75 (1.26–2.43)], those with AMI [HR (95% CI) 1.61 (1.10–2.36)], and those without AMI [HR (95% CI) 2.04 (1.06–3.91)] (all *P* < 0.05). Interestingly, the predictive value of the TyG index seemed to be more prominent in patients with BMI < 30 kg/m^2^ [HR (95% CI) BMI < 30 kg/m^2^ 2.30 (1.54–3.44) vs. BMI ≥ 30 kg/m^2^ 0.95 (0.54–1.69), *P* for interaction = 0.003] and without diabetes [HR (95% CI) without diabetes 2.09 (1.41–3.08) vs. with diabetes 1.26 (0.71–2.23), *P* for interaction = 0.037] (Fig. 5). Similar results were obtained in stratified analyses of the TyG index and ICU mortality (Additional file 3: Fig. S2).

## Discussion

To our knowledge, this study is the first to explore the relationship between the TyG index and prognosis in critically ill patients with CHD from a United States (US) cohort. The main finding of the study was that an elevated TyG index was a strong independent predictor of greater mortality in critically ill patients with CHD, and this finding persisted after adjustment for possible confounders. In addition, our study revealed that the TyG index was significantly linearly associated with the risk of all-cause mortality in critically ill patients with CHD.

### Insulin resistance, TyG index, and CVD risk

Despite guideline-directed therapy, CHD remains the primary cause of death globally [16]. Hyperglycemia in patients admitted to the ICU with AMI is commonly observed and is caused by the release of various cytokines from the damaged heart muscle [17, 18]. This cardiogenic insulin resistance has both cardiac and systemic effects [19]. The major independent roles of the cardiac insulin resistance in the risk of post myocardial infarction and related complications have been highlighted [20]. IR is associated with dysmetabolic conditions, and is not only a risk factor for the development of CVD but also may affect adverse cardiovascular outcomes [21]. The hyperinsulinemic-euglycemic clamp technique is the gold standard for assessing IR, whereas Homeostatic Model Assessment of IR (HOMA-IR) is the most widely used method [22]. However, the hyperinsulinemic-euglycemic clamp technique is costly, time‐consuming, and invasive [23], whereas HOMA-IR constrained to the requirement of insulin concentrations examination, which are not routinely measured in clinical practice [24]. In this regard, researchers began to study the TyG index, and found that it was a reproducible, reliable, cost-effective, and valid surrogate marker of IR [25]. A previous study has shown that the TyG index is highly sensitive (96.5%) and specific (85.0%) for the detection of IR, as compared with the hyperinsulinemic–euglycemic clamp technique [26]. Furthermore, the TyG index has been demonstrated to have better performance than HOMA-IR [27]. Because glucose and TG tests are available in all clinical laboratories, the TyG index can be widely used in clinical practice.

### Related studies

Numerous clinical studies have been conducted to investigate the association of the TyG index with CVD morbidity and mortality in the general population and many patient cohorts. Park et al. [28] have found that the TyG index was an independent marker of the presence of CHD, particularly non-calcified or mixed plaques, in asymptomatic individuals without traditional cardiovascular risk factors. In a large-scale cohort of participants who underwent regular health check-ups, higher TyG index values were significantly associated with greater risk of CVD, including fatal and non-fatal CHD [29]. Similarly, another study that enrolled 5014 apparently healthy individuals has indicated that a higher TyG index was significantly associated with greater risk of incident CVD, independently of other known cardiovascular risk factors [30]. And for patients with stable coronary artery disease, TyG index has been demonstrated to be positively related to future cardiovascular events, suggesting that TyG may be a useful marker for predicting clinical outcomes in patients with coronary artery disease [31]. Zhou et al. [32] demonstrated that TyG index representing IR was associated with an increased risk of stroke recurrence, all-cause mortality, and neurologic worsening in patients with ischemic stroke. In addition, a study with more than 10 years of follow-up has found that the TyG index (as a surrogate for IR) was a significant risk factor for incident CVD/CHD with an issue that was more prominent among the younger population [33]. Moreover, the findings of Sun et al. [34] showed that TyG index was associated with all-cause mortality and cause-specific mortality (CVD and malignant neoplasms) among middle age and elderly US population. These data support the utility of the TyG index as a reliable and valid marker of IR for risk stratification in the real world.

### Comparison with other studies and what does the current work add to the existing knowledge

Current data about associations between TyG index and critically ill patients are limited. Recently, Zhai et al. [35] found that TyG index was a strong indicator of in-hospital mortality in critically ill patients with heart disease (including congestive heart failure, arrhythmias, coronary artery disease, acute coronary syndrome, valve disease, and cardiomyopathy). Additionally, our study was the first to demonstrate that the TyG index was a strong independent predictor of hospitalization and ICU mortality in critically ill patients with CHD. Though, Zhai et al. [35] reported that TyG index linked to in-hospital mortality in critically ill patients with heart disease. However, in this specific cohort of ICU patients with CHD, we found that TyG index was a strong independent predictor of greater mortality in these patients. Most importantly, for CHD, a global disease with high morbidity and high mortality, our findings will allow for early identification of patients with high residual risk, which is essential for better clinical management to reduce future adverse events.

Intriguingly, in our study, approximately seven-tenths of the patients had AMI; however, our subgroup analysis showed that the predictive value of the TyG index was consistent in patients with and without AMI. Therefore, the predictive value of the TyG index for mortality in CHD patients remained consistent regardless of whether the patient was admitted to the ICU for AMI. However, we did not find any association between the TyG index and in-hospital all-cause mortality in participants with diabetes or CKD at baseline. This outcome might be explained by reverse causality: patients previously diagnosed with these illnesses generally might have been under treatment or might have adopted healthier habits; thus, their analytical parameters might have been well controlled despite their high risk of all-cause mortality [30]. In addition, the present study revealed that the predictive value of IR, as indicated by the TyG index, seemed to be more prominent in patients with BMI < 30 kg/m^2^ [HR (95% CI) BMI < 30 kg/m^2^ 2.30 (1.54–3.44) vs. BMI ≥ 30 kg/m^2^ 0.95 (0.54–1.69), *P* for interaction = 0.003]. In contrast, a previous study has reported that the predictive value of the TyG index is more prominent in patients with BMI > 28 kg/m^2^ [36]. This discrepancy might be associated with differences in participant selection across studies. Further research is needed to validate the relationship between the TyG index and BMI.

In addition, despite the IR was not a traditional risk factor of CHD [37]. However, in the present study, TyG index levels were positively associated with BMI, FBG, HbA1c, TG, total cholesterol, and LDL, and were negatively associated with HDL, suggesting that the observed association between the TyG index and unfavorable prognosis may be explained by the presence of traditional risk factors of CHD. Consistent with previous studies [38]. Moreover, higher TyG index quartiles were associated with the increased length of ICU stay and hospital stay, as well as higher hospital and ICU mortality, which undoubtedly impose a heavy burden on families and society. Therefore, the prognosis of critically ill patients requires greater attention, and potential risk factors contributing to this residual cardiovascular risk must be identified to improve healthcare for this population.

### Possible mechanisms

Although the exact biological mechanisms accounting for the relationship between TyG index and mortality remain unclear, the possible crucial pathway may be associated with IR. IR is a state of decreased sensitivity and responsiveness to the action of insulin. Individuals with IR are predisposed to the development of several metabolic disorders, such as hyperglycemia, dyslipidemia, and hypertension, all of which are strongly associated with poor CVD outcomes [39]. The chronic hyperglycemia and dyslipidemia induced by IR can trigger oxidative stress, aggravate inflammatory responses, enhance foam cell formation, impair endothelial function, and promote smooth muscle cell proliferation [40]. Moreover, hyperinsulinemia can increase sympathetic nervous system activity and renal sodium retention. Persistent IR can raise the blood pressure, increase cardiac burden, and lead to vascular and renal damage [41]. All these pathophysiological changes can further lead to the initiation and progression of CHD, thereby resulting in poor prognosis. Study have shown that FBG mainly reflects IR from liver, whereas fasting TG mainly reflects IR from adipose cells. Therefore, the TyG index may reflect IR from two aspects and thus be closely associated with IR [36].

### Study strengths and limitations

The main strength of our study was that we confirmed that an increase in the TyG index was a strong independent predictor of greater mortality in critically ill patients with CHD admitted to the ICU in a US cohort. However, this study also had several limitations. First, this was a single-center retrospective study and therefore could not definitively establish causality. Despite multivariate adjustment and subgroup analyses, residual confounding factors might have affected the prognosis. Second, we did not compare the risk of all-cause mortality between patients treated with percutaneous coronary intervention and coronary artery bypass grafting, owing to the limited number of studies. Finally, our analysis focused only on the prognostic value of the baseline TyG index in CHD. However, the TyG index might have changed during the hospital stay; hence, further research is needed to verify whether the change in the TyG index also predicts mortality.

## Conclusions

Together, our results extended the utility of the TyG index to critically ill patients with CHD, and demonstrated that the TyG index was a potential predictor of hospital and ICU mortality among these patients. Moreover, the TyG index was significantly linearly correlated with the risk of all-cause mortality in critically ill patients with CHD. Measuring the TyG index could contribute to risk stratification and prognosis prediction in patients with CHD. Further studies are required to determine whether interventions focused on the TyG index improve clinical prognosis in this population.

## Supplementary Information

**Additional file 1: Table S1.** Missing number for risk variables and outcome variables.

**Additional file 2: Figure S1.** Hazard ratios (95% CIs) for ICU mortality according to TyG index quartiles after adjusting for age, sex, BMI, dyslipidemia, hypertension, diabetes, chronic kidney disease, respiratory failure, white blood cell, red blood cell, hemoglobin, serum creatinine, SIRS score.

**Additional file 3: Figure S2.** Forest plots of hazard ratios for the primary endpoint in different subgroups.

## Data Availability

The datasets generated and analyzed during the current study are available from the corresponding author on reasonable request.

## Abbreviations

CHD: Coronary heart disease
ICU: Intensive care unit
IR: Insulin resistance
TyG: Triglyceride-glucose
MIMIC-III: Medical information mart for intensive care III
TG: Triglyceride
SQL: Structured query language
BMI: Body mass index
SOFA: Sequential organ failure assessment
SIRS: Systemic inflammatory response syndrome
APSIII: Acute physiology score III
SAPSII: Simplifed acute physiological score II
AKI: Acute kidney injury
CKD: Chronic kidney disease
AMI: Acute myocardial infarction
MI: Myocardial infarction
KDIGO: Kidney disease: improving global outcomes
Scr: Serum creatinine
HR: Hazard ratio
CI: Confidence interval
WBC: White blood cell
RBC: Red blood cell
FBG: Fasting blood glucose
LDL: Low-density lipoprotein
HDL: High-density lipoprotein
HbA1c: Hemoglobin A1c
CVD: Cardiovascular disease
US: United States
HOMA-IR: Homeostatic model assessment of IR
