# Supplementary material for: Independent effects of the triglyceride-glucose index on all-cause mortality in critically ill patients with coronary heart disease: analysis of the MIMIC-III database
Source: Cardiovasc Diabetol. 2023 Jan 13;22:10. doi: 10.1186/s12933-023-01737-3 (PMC9838037; doi:10.1186/s12933-023-01737-3)
Supplement: Supplementary file 1 — Additional file 1: Table S1. Missing number for risk variables and outcome variables. [file 12933_2023_1737_MOESM1_ESM.docx]

**Table S1. Missing number for risk variables and outcome variables**

| **Risk Variables** | Missing number (%) |
| --- | --- |
| Age | 0 (0) |
| Gender, male | 0 (0) |
| BMI | 389 (24.04%) |
| SOFA score | 0 (0) |
| SIRS score | 0 (0) |
| APSIII | 0 (0) |
| SAPSII | 0 (0) |
| Heart failure | 0 (0) |
| Hypertension | 0 (0) |
| Atrial fibrillation | 0 (0) |
| Dyslipidemia | 0 (0) |
| Diabetes | 0 (0) |
| Respiratory failure | 0 (0) |
| AKI | 0 (0) |
| CKD | 0 (0) |
| AMI | 0 (0) |
| PCI | 0 (0) |
| CABG | 0 (0) |
| WBC | 0 (0) |
| Lymphocyte | 398 (24.60%) |
| Neutrophil | 397 (24.54%) |
| RBC | 0 (0) |
| Hemoglobin | 0 (0) |
| Platelet | 0 (0) |
| Serum potassium | 0 (0) |
| Serum sodium | 0 (0) |
| TC | 61 (3.77%) |
| TG | 0 (0) |
| LDL | 74 (4.57%) |
| HDL | 68 (4.20%) |
| HbA1c | 340 (21.01%) |
| Glucose | 0 (0) |
| Albumin | 355 (21.94%) |
| UCr | 388 (23.98%) |
| Scr | 0 (0) |
| BUN | 0 (0) |
| TyG index | 0 (0) |

**Abbreviations:** BMI, body mass index; SOFA, sequential organ failure assessment; SIRS, systemic inflammatory response syndrome; APSIII, Acute physiology score III; SAPSII, Simplifed acute physiological score II; AKI, acute renal injury; CKD, chronic kidney disease; AMI, acute myocardial infarction; PCI, percutaneous coronary intervention; CABG, coronary artery bypass grafting; WBC, white blood cell; RBC, red blood cell; TC, total cholesterol; TG, triglyceride; LDL, low-density lipoprotein; HDL, high-density lipoprotein; HbA1c, hemoglobin A1c; UCr, urine creatinine; SCr, serum creatinine; BUN, blood urea nitrogen; TyG index, triglyceride glucose index.
