# Supplementary figures and images for: Independent effects of the triglyceride-glucose index on all-cause mortality in critically ill patients with coronary heart disease: analysis of the MIMIC-III database
Source: Cardiovasc Diabetol. 2023 Jan 13;22:10. doi: 10.1186/s12933-023-01737-3 (PMC9838037; doi:10.1186/s12933-023-01737-3)

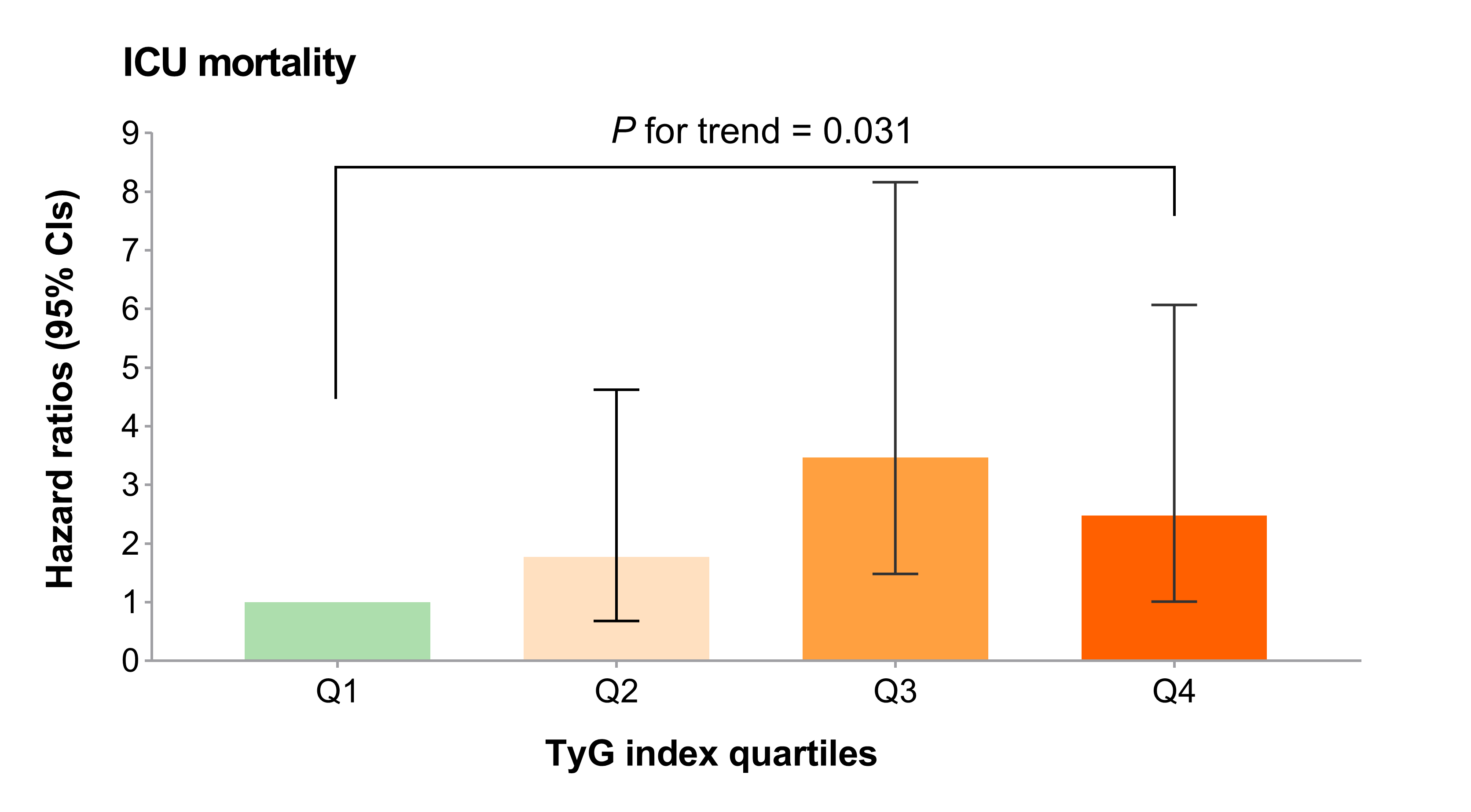

Supplement: Supplementary file 2 — Additional file 2: Figure S1. Hazard ratios (95% CIs) for ICU mortality according to TyG index quartiles after adjusting for age, sex, BMI, dyslipidemia, hypertension, diabetes, chronic kidney disease, respiratory failure, white blood cell, red blood cell, hemoglobin, serum creatinine, SIRS score. [file 12933_2023_1737_MOESM2_ESM.png]

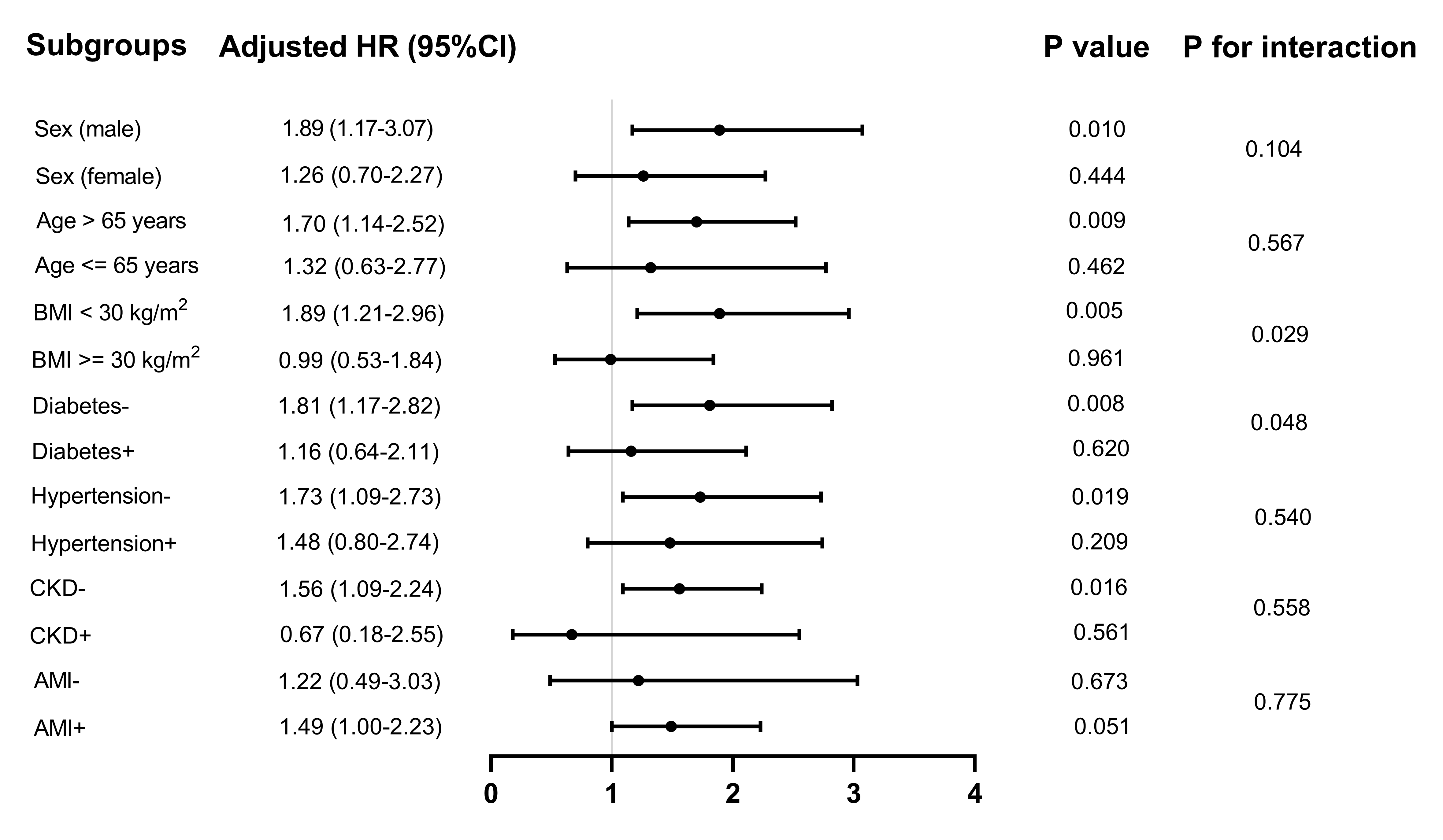

Supplement: Supplementary file 3 — Additional file 3: Figure S2. Forest plots of hazard ratios for the primary endpoint in different subgroups. [file 12933_2023_1737_MOESM3_ESM.png]
